# Cystic Adventitial Disease of the Popliteal Vein, a Rare Cause of Lower Limb Deep Vein Thrombosis

**DOI:** 10.1016/j.ejvsvf.2022.02.002

**Published:** 2022-02-10

**Authors:** Ricardo Correia, Nuno Gião, Rita Bento, Rita Garcia, Nelson Camacho, Maria E. Ferreira

**Affiliations:** a Angiology and Vascular Surgery Department, Hospital de Santa Marta, Centro Hospitalar Universitário Lisboa Central, Portugal; b Pathological Anatomy Department, Hospital de São José, Centro Hospitalar Universitário Lisboa Central, Portugal

**Keywords:** Cyst, Patch popliteal venoplasty, Popliteal vein, Venous cystic adventitial disease (CAD)

## Abstract

**Introduction:**

Cystic adventitial disease (CAD) is characterised by the accumulation of gelatinous fluid within the adventitial layer of a blood vessel. Over 90% of CAD occurs in the arterial system. Venous CAD most commonly involves the iliofemoral rather than the popliteal segments.

**Report:**

This is the report of a 49 year old female patient with a previous right leg deep vein thrombosis (DVT). She presented to a vascular outpatient appointment with recurrent right lower extremity swelling. Venous duplex ultrasound showed an ectatic and incompetent right popliteal vein. Computed tomography (CT) venography showed focal ectasia of the right popliteal vein resulting from an eccentric low density cyst with a diameter of 15 mm. Under general anaesthesia, the patient was placed in the prone position. A lazy S incision was performed in the right popliteal fossa. The popliteal vein had an eccentrically thickened lateral bulge. After heparinisation, a longitudinal venotomy, endophlebectomy, and *en bloc* cyst removal were performed sequentially. Popliteal patch venoplasty was performed subsequently using the ipsilateral small saphenous vein. After six months, the patient remains on rivaroxaban. A follow up venous duplex ultrasound showed vein reflux through a standard calibre popliteal vein without evidence of cyst recurrence.

**Conclusion:**

Venous CAD is a rare disease and should be considered if previous DVT or symptoms mimicking DVT occur. Cyst resection and reconstruction with vein patch, venous or synthetic graft is the most commonly used strategy and has lower rates of cyst recurrence and need for re-operation.

## Highlights

• Venous cystic adventitial disease is rare.
• Adventitial vein cysts may cause oedema and vein thrombosis.
• Venous ultrasound is the first diagnostic test.
• Complete cyst wall excision is crucial to prevent recurrence.

## Introduction

Cystic adventitial disease (CAD) is characterised by accumulation of gelatinous fluid containing mucoproteins and mucopolysaccharides within the adventitial layer of a blood vessel.^1^^,^^2^ Over 90% of CAD occurs in the arterial system.^1^^,^^2^ However, the true incidence of venous CAD is probably underestimated because it can be asymptomatic or misdiagnosed as deep vein thrombosis (DVT).^3^, ^4^, ^5^ Venous CAD most commonly involves the iliofemoral rather than the popliteal segments.^6^ Popliteal vein (POPV) cysts comprise only 0.5% of all cystic adventitial disease cases and 7% of venous cases.^1^^,^^2^^,^^7^

The aetiology and pathogenesis of CAD remain controversial. The theories reported mainly refer to arterial CAD, and the one most accepted states that articular capsular defects allow synovial fluid to leak into the adventitia of the capsular vessel.^8^ Like arterial CAD, men are more commonly affected in vein CAD, although to a lesser degree, with a male to female ratio of 2.3: 1.^9^

The cyst can lead to lumen occlusion and cause various symptoms and signs depending on the vein location.^1^ Clinical presentation may vary from limb swelling or groin mass to local compressive symptoms.^4^^,^^10^ Typically, the diagnosis is made when a young, previously healthy male presents with painless asymmetric lower extremity oedema and is investigated for DVT.^5^^,^^6^^,^^9^

A case of popliteal venous CAD treated by cyst resection and saphenous vein patch repair is described.

## Case description

Five years ago, a 49 year old woman patient had a right femoropopliteal DVT treated with rivaroxaban and compressive therapy for six months.

Without any regular medication she presented to a vascular outpatient appointment with recurrent painless right below knee swelling and fatigue. The symptoms were worse in the summer months and at the end of the working day.

A previously performed outpatient clinic venous duplex ultrasound (DUS) reported an ectatic and incompetent right POPV with a lateral bulge without Doppler signal.

**Figure 1:**
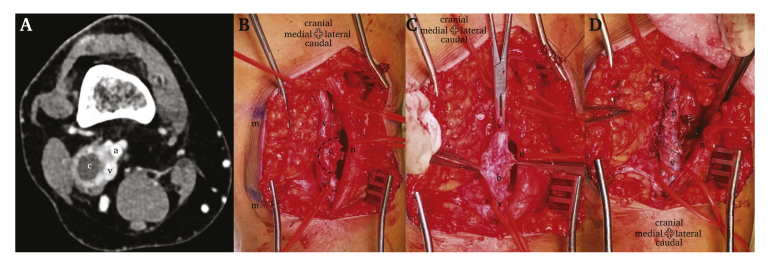
Popliteal vein cyst imaging and removal: computed tomography venography, A; popliteal vein exposure, B; popliteal vein after venotomy. C; patch popliteal venoplasty, D (a, popliteal artery; c, cyst; m, skin marks; n, nerve; p, venous patch; v, popliteal vein).

Computed tomography (CT) venography (Fig. 1A) showed focal ectasia of the right POPV (25mm maximum calibre) with an eccentric low density cyst with a diameter of 15 mm; the vein lumen was patent but slightly narrowed.

The patient gave informed consent for the planned procedure and use of anonymised data and images for scientific purposes.

### Operative procedure

Under general anaesthesia, the patient was placed in the prone position. After careful ultrasound marking of the diseased vein segment borders, a lazy S incision in the right popliteal fossa was performed. Dissection was carried out in the popliteal fossa through moderately inflammatory reactive tissue. The POPV had an eccentric thickened lateral bulge without a typical fluid filled transparent cyst (Fig. 1B). There was no Baker's cyst or synovial connection. After systemic and local heparinisation, the POPV was clamped and a longitudinal venotomy adjacent to the bulging was performed; the vein lumen was patent but filled with thickened fibrous bands (Fig. 1C). *En bloc* cyst removal (that included both the cyst and the adjacent vein wall) and endophlebectomy were performed. Popliteal patch venoplasty with ipsilateral small saphenous vein and a continuous 6-0 polypropylene suture completed the procedure (Fig. 1D).

### Post-operative course

The post-operative period was uneventful, and the patient was discharged on the first post-operative day taking 20 mg rivaroxaban daily. Compression below knee stockings (20 mmHg at the ankle) were used day and night for the first two post-operative days and during the day thereafter.

**Figure 2:**
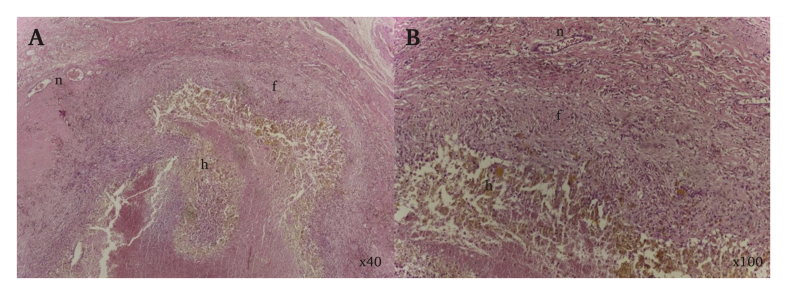
Histology of the vessel wall with cystic degeneration with histiocytes with haemosiderin pigment (h), fibrosis (f) and neovessels (n) – Panel A HE ×40; Panel B HE ×100.

Pathological examination of the resected specimen showed a vessel wall with areas of hyalinised fibrosis and cystic degeneration, with neovessels and chronic inflammatory infiltrate (Fig. 2).

Six months after surgery, the patient described persistent below knee swelling at the end of the day. Follow up venous DUS showed vein reflux through a standard calibre POPV with no evidence of cyst or DVT recurrence. The plan is to keep the patient on therapeutic oral anticoagulant and daily compression stockings until two years after surgery if the clinical and DUS biannual evaluation reveal the absence of DVT recurrence.

## Discussion

Popliteal venous CAD is quite rare. So far, most of the literature published on venous CAD reports cysts in the common femoral vein (65%) and external iliac vein (18%) without previous DVT (93%)^7^ (Table 1). The authors present the effective surgical treatment of a patient with a POPV cyst as the likely cause of previous femoropopliteal DVT.

**Table 1:** Previously published popliteal vein cysts case reports.

Patient	Main author	Year of publication	Clinical presentation	Previous DVT	Artery	Treatment	Vein reconstruction	Synovial extension
1	Ikeda	1984	Leg easy fatigability	No	Compressed	Cyst excision	No	Yes
2	Sakamoto	2006	Leg swelling	No	Not affected	Cyst excision	No	No
3∗	Lee	2018	Leg swelling	Yes	Focal occlusion	Cyst excision and popliteal artery reconstruction	No	No
4	Chan	2021	Foot numbness	No	Not affected	Cyst excision	Patch venoplasty	No

CAD = cystic adventitial disease; DVT = deep vein thrombosis; POPV = popliteal vein.

∗ This patient had popliteal artery CAD, but the large cyst compressed the POPV.

In the setting of unilateral lower extremity oedema, venous DUS should be the first diagnostic test. An experienced sonographer can reliably visualise a cystic mass in the blood vessel wall and evaluate the number and dimensions of the cysts and any associated narrowing of the vein lumen. Even if not diagnostic, the DUS may suggest the diagnosis of CAD, as was the case in this patient.^6^^,^^9^ CT has the ability to demonstrate the size of the cysts and their relationship to surrounding structures. As in the present case, it helps to plan treatment.^4^^,^^6^ At some institutions, MRI is used for the workup of CAD. MRI depicts the extent of cystic involvement with increased tissue definition, and many authors consider it essential during the planning of surgical intervention.^1^^,^^6^^,^^9^ Because of the high cost and limited availability, it was not performed in this case. Nevertheless, it should be acknowledged that venous CAD is not always diagnosed before surgery.

Macroscopically, cysts are either unilocular (70.4%) or multilocular (29.6%) and filled with clear or yellow mucoid material between the media and adventitia. As fluid accumulates within the cyst, it compresses the vein lumen, resulting in stenosis or occlusion.^6^ Grossly, the POPV may appear enlarged and connected by adhesions to adjacent structures. At the time of operation, these cysts are apparent following incision of the adventitial layer.^4^^,^^6^ This explains why dissection of the popliteal fossa may be difficult, as the cyst is not always well delineated.

Because only a small number of cases of venous CAD have been reported and there is a lack of consensus regarding the aetiology, the optimal treatment for the condition is unknown.^2^ Although some authors reported successful non-invasive management of venous CAD (cyst aspiration with or without sclerosant, and cyst drainage), open surgical management remains the standard treatment for this condition. Surgical techniques have resulted in different rates of symptom control and recurrence in the literature.^2^ The most crucial objective of treatment is complete excision of the cyst wall, which prevents the mucoid cyst's mesenchymal cells from continuing to secret enough mucin to result in cyst recurrence. This preferred strategy was used in this case. It should be attempted first if technically feasible because it has the most favourable long term outcomes with much lower rates of cyst recurrence and need for re-operation.^2^^,^^4^

In this patient, with venous CAD combined with previous venous thrombosis, there was wall thickening and persistent venous stenosis. In such cases, it is recommended to resect the involved vein segment completely followed by graft reconstruction with either an interposed vein or synthetic graft. A valid alternative consists of performing endophlebectomy in combination with cyst excision and saphenous vein patch angioplasty, as was performed in this case. In this way, a major deep vein reconstruction could be avoided.^3^^,^^6^^,^^9^

Duplex scanning is a useful post-operative surveillance tool to exclude cyst recurrence and residual or recurrent stenosis.^6^ In this patient, it also monitored DVT recurrence.

The optimal duration of anticoagulation after cyst associated DVT or vein surgery is not defined. The increased recurrence risk after unprovoked DVT is higher, and it may justify extended anticoagulation.

In conclusion, venous CAD is a rare disease and should be considered in cases of previous DVT or if symptoms and signs mimic DVT. Cyst resection and reconstruction with vein patch, venous, or synthetic graft is the most commonly used strategy.

## Conflict of interest

None.

## Funding

None.
