# Supplementary material for: Cystic Adventitial Disease of the Popliteal Vein, a Rare Cause of Lower Limb Deep Vein Thrombosis
Source: EJVES Vasc Forum. 2022 Feb 10;54:75–8. doi: 10.1016/j.ejvsvf.2022.02.002 (PMC8921300; doi:10.1016/j.ejvsvf.2022.02.002)
Supplement: Multimedia component 1 [file mmc1.pdf]

Supplementary references

11. Ikeda M, Fujimori Y, Tankawa H, Iwata H. Compression syndrome of the popliteal vein and artery caused by popliteal cyst. Angiology 1984; 35(4):245-51;

12. Sakamoto A, Tanaka K, Matsuda S, Harimaya K, Nakamura T, Oda Y *et al*. Adventitial Cystic Disease of the Popliteal Vein: Report of a Case. Surg Today 2006; 36:1098–1100;

13. Lee JS, Hwang JK, Park SC, Sang Kim D. Surgical Management of Symptomatic Adventitial Cystic Disease of the Popliteal Artery as a Cause of Deep Vein Thrombosis. Chin Med J 2018; 131:2141-2;

14. Chan MCY, Cornwall J, Ilonzo N, McKinsey J. Cystic adventitial disease of the popliteal vein and artery in siblings. J Vasc Surg Cases and Innovative Techniques 2021; 7:545-8;

15. Lun Y, Zhang J, Jiang H, Xu D, Sun J, Wang S *et al*. Treatment Options for Venous Cystic Adventitial Disease: A Case Report and Literature Review. Ann Vasc Surg 2020; 64:413.e1–413.e4;

16. Kakkos SK., Gohel M, Baekgaard N, Bauersachs R, Bellmunt-Montoya S, Black SA *et al*. European Society for Vascular Surgery (ESVS) 2021 Clinical Practice Guidelines on the Management of Venous Thrombosis. Eur J Vasc Endovasc Surg 2021; 61:9-82.
